# A Federated Data Linkage Strategy to Support Population Health Research in Canada

**DOI:** 10.23889/ijpds.v1i1.388

**Published:** 2017-04-19

**Authors:** Tanya Flanagan, Céline Moore, Carolyn Sandoval

**Affiliations:** 1 Canadian Partnership Against Cancer

## Objectives

Canada has established a pan-Canadian cohort with over 300,000 volunteer participants aged 35-69, to support research on cancer and chronic disease. A key feature of the cohort is that participants have consented to link their cohort data with administrative datasets. This prospective cohort, representing nearly 1 in 50 Canadians in this age range will be followed for multiple decades, building a platform that supports access to timely, high-quality, data related to cancer and other chronic diseases, which will enable researchers to answer complex system questions and achieve better health outcomes for Canadians.

## Approach

A baseline "core" questionnaire was administered to participants capturing information on socio-demographics, economic characteristics, personal and familial history of diseases and lifestyle and health behaviours. A re-contact questionnaire is planned for 2016 to update baseline information and add depth to specific areas and capture changes over time. To realize the full potential for this cohort to support transformative research it is crucial to be able to link this data with other provincial datasets, such as cancer registries, hospital records and mortality data.

For the most part, health data in Canada resides under the purview of health providers and, or government custodians in each of the provinces and territories. As such, an innovative federated data linkage strategy is required to link cohort data with health administrative data in each regions, adhering to existing privacy and regulatory requirements, while providing central access for researchers.

## Results

To date, 40% of all cohort participants have been linked with priority provincial administrative data. Each province has its own unique data linkage challenges, requiring unique customized solutions. By the end of 2017 we anticipate that the number of participants who will have had their data linked will increase to nearly 70%. The federated data linkage strategy and infrastructure offer an innovative approach that others can learn from; however to realize the full potential of the cohort and support transformative research partnerships and collaborations are required.

## Conclusion

Efforts to organize resources and establish systems for data linkage and optimize data sharing and utilization in Canada are underway and include discussions with the provincial privacy commissioners, national and provincial and territorial data custodians and other thought leaders in the field.

